# Explore barriers and risks that hinder AI integration into global talent management: A Systematic Review

**DOI:** 10.12688/f1000research.179429.1

**Published:** 2026-05-25

**Authors:** Godfrey Maake, Adele Bezuidenhout, Cecilia. M Schultz, Pieter K Smit

**Affiliations:** 1 BIMS, Tshwane University of Technology, Pretoria, Gauteng, South Africa; 2 School of Organisations, People and Systems, University of Portsmouth, Portsmouth,, UK; 3 Department of People Management and Development, Tshwane University of Technology, Pretoria, Gauteng, South Africa; 4 Department of People Management and Development, Tshwane University of Technology, Pretoria, Gauteng, South Africa

**Keywords:** Artificial intelligence, global, talent management; Human resource management

## Abstract

The work environment is rapidly changing due to the constantly rising global disruptions. It has therefore become essential for organisations to retain their top talent in order to remain sustainable. This systematic review aimed to identify the primary barriers and risks associated with implementing Artificial Intelligence (AI) in Global Talent Management (GTM). A systematic literature review was undertaken in accordance with PRISMA-ScR standards. The review searched eight databases (Google Scholar, Scopus, Web of Science, ScienceDirect, ProQuest, Emerald Insight, Sabinet, and Elsevier) using a comprehensive search approach. Articles published between 2020 and 2025 in peer-reviewed journals were considered. The entire texts of selected studies were assessed against the inclusion criteria, and 23 peer-reviewed empirical studies were included in the full review. An analysis of 23 scholarly articles identified three key barriers and risks to implementing AI within GTM, including governance and accountability, implementation hurdles, and ethical and technical concerns. The successful integration of AI into GTM depends on more than just technology. It requires a confluence of strategic vision, enabling infrastructure, cultural readiness, and ethical oversight. The risks associated with AI reflect a failure to align innovation with governance, human-centred design, and long-term trust-building.

## Introduction

The work environment is rapidly changing due to the constantly rising global disruptions. It has therefore become essential for organisations to retain their top talent in order to remain sustainable. In response to the fast-paced changes in the global business landscape and the growing demand for a capable and adaptable workforce, talent management has taken centre stage in modern human resource practices ( Tsaousiotis, Panitsidis, Spinthiropoulos & Zafeiriou, 2025). On the other hand, there is no doubt that attracting, developing, and retaining talent has become one of the most pressing challenges for organisations worldwide ( Gallardo-Gallardo, Thunnissen, & Scullion, 2020). To achieve organisational strategic goals, organisations require forward-thinking, high-potential employees ( Boštjančič & Slana, 2018). Talent management refers to the comprehensive process of attracting, training, developing, evaluating, and retaining an organisation’s most valuable asset—its people ( Sareen & Mishra, 2016). It is a strategic initiative focused on identifying and nurturing skilled individuals to ensure long-term organisational success ( Ekhsan, Parashakti, & Perkasa, 2023). Consequently, organisations should prioritise a robust talent management strategy to remain competitive in the working landscape ( Sindhura, 2022).

As the workplace becomes increasingly complex, effective talent management has emerged as a key driver of competitive advantage. Undeniably, it remains a fundamental component of human resource management, which includes attracting employees, developing the existing employees, motivating and retaining high-performing employees ( Zake, Jonck & Pelser, 2024; Sindhura, 2022). The role of Human Resource Management (HRM) has rapidly transformed from handling basic administrative tasks, such as hiring, payroll, and employee benefits, to embracing a more strategic focus that supports and advances organisational goals ( Amushila & Bussin, 2021). Globally, organisations are increasingly taking a proactive approach to attract talented individuals who can foster growth, spark innovation, and adapt to evolving market needs ( Tsaousiotis, Panitsidis, Spinthiropoulos & Zafeiriou, 2025). As technology advances rapidly, artificial intelligence (AI is increasingly integrated into the core functions of HRM ( Bujold, Roberge-Maltais, Parent-Rocheleau, Boasen, Sénécal, & Léger, 2024). The integration of AI in human resource functions will automate workflows and tasks ( Babashahi, Barbosa, Lima, Lyra, Salazar, Argôlo, Almeida, & Souza, 2024). However, by embracing this transition with vision, leadership, and human-centred strategies, Human Resource Development (HRD) professionals can steer their organisations toward an empowering AI future, where both human potential and AI capabilities are congruently elevated ( Ghosh, Nachmias, Murdoch, & McGuire, 2024). While some fear that AI will replace human workers, others believe it has the potential to supplement and enhance human labour by increasing productivity, efficiency, and innovation ( Spulbar & Mitrache, 2025).

Employee adoption of AI is crucial because it affects both the efficiency of AI implementation and its effectiveness in improving job performance ( Ghaleche, 2023). Although AI has transformed specific sectors, its capacity to reshape Human Resource (HR) functions across the board is equally compelling ( Ghosh, Nachmias, Murdoch & McGuire, 2024). To fully grasp the transformative impact of AI within the human resources domain, it is essential to explore the key factors driving its integration into GTM.

The structure of the article is as follows: Section 1 introduces the study and provides background information; Section 2 reviews relevant literature; Section 3 outlines the methodology used for selecting and analysing articles; Section 4 presents the study’s findings; Section 5 discusses these findings, organized into themes and subthemes; and Section 6 offers the overall conclusions, including management implications, limitations, and suggestions for future research.

### Problem statement

AI has the potential to improve talent management techniques through the implementation of advanced automated workforce management solutions ( Faqihi & Miah, 2023). AI has transformed modern workplaces in an unprecedented manner, leading to digital workstyles in HRM ( Singh & Pandey, 2024). While the integration of AI in businesses has potential, it also presents problems that must be addressed to achieve successful deployment ( Scheffknecht, 2025). AI has demonstrated its promise in supporting human decision-making, but there are challenges to using AI for this purpose ( Booyse & Scheepers, 2024). The integration of AI in corporate contexts is rapidly increasing; however, substantial limitations prevent its successful application and adoption ( Alawamleh, Shammas, Alawamleh, & Ismail, 2024). Organisations often face difficulties when integrating AI into HR processes, primarily due to limited resources such as skilled IT professionals and adequate technological infrastructure ( Madanchian & Taherdoost, 2025). A shortage of experienced AI specialists affects implementation efforts, underscoring the importance of developing skills and competence ( Zavodna, Überwimmer, & Frankus, 2024). Issues such as algorithmic bias, the dehumanisation of work relationships, the opacity of automated decision-making systems, and job displacement have sparked heated debate, underscoring the importance of a responsible and thoughtful approach ( Rosário, 2025; Madanchian & Taherdoost, 2025). Mwita and Kitole conducted their investigation in 2025 and identified substantial obstacles, including biases towards a lack of transparency, privacy concerns, and the inability to address the qualitative components of HR processes. Although numerous studies have explored talent management and AI, a notable gap remains in scholarly research examining the barriers and risks that hinder AI adoption in GTM. Therefore, this study seeks to close this gap.

### Research gap and purpose

This systematic review aimed to identify the primary barriers and risks associated with implementing AI in GTM.

## Literature review

### Concept of AI

AI is considered a key element of the Fourth Industrial Revolution, which the organisations believe will transform the way businesses function. With the rapid advancement of AI technologies, their incorporation into various work domains has become unavoidable ( Tiwari, Babu, Marda, Mishra, Bhattar, & Ahluwalia, 2024). AI is rapidly transforming the global workforce and corporate environment through task automation, enhanced decision-making, and the facilitation of innovative operational methods ( Farouk, 2025). It encompasses a collection of technologies that perform computational functions often designated for humans and is crucial to contemporary global discussions regarding social and technical transformations ( Benhamou, 2020). What has become a standard practice in organisations across the globe is the integration of AI into business operations to perform routine low level tasks that were performed by humans ( Poisat, Cullen & Calitz, 2024). It is defined as computer systems capable of learning and executing activities generally associated with human intelligence, with the potential to ultimately surpass human capabilities ( Diyin, Bhaumik, & Wang, 2024). AI employs algorithms that combine high-quality data and fast computational services, resulting in Core AI, which provides stability and precision for everyday activities ( Bhardwaj, Singh, and Kumar, 2020). As a result, there is little doubt that incorporating AI into organisational work practices brings considerable advantages, leading to meaningful enhancements in various areas of business operations ( Murire, 2024). AI is designed to streamline, automate, and support decision-making within organisations, because it complements the employee’s tasks ( Tiwari, Babu, Marda, Mishra, Bhattar, & Ahluwalia, 2024). As noted by Rožman, Oreški, and Tominc (2022), AI enhances operational efficiency and creates opportunities for the development of new products. AI has the potential to enhance employee engagement by customising opportunities for professional growth and increasing job satisfaction ( Diyin, Bhaumik, & Wang, 2024).

### Integration of AI in human resources

Human Resource Management (HRM) plays a pivotal role in organisations, encompassing key functions such as recruitment, employee retention, and motivation ( Ochieng, 2023). Traditional HRM approaches have faced criticism for being time-intensive and influenced by human bias, subjectivity, and personal judgment ( Bujold et al., 2024). The adoption of AI in HRM has transformed organisational functions, reflecting its ability to analyse candidate data more quickly while remaining free of human prejudice or inaccuracy ( Liu, Li, & Xia, 2021; Rosário, 2025; Pandey & Khaskel, 2019). AI has undeniably become a valuable asset in handling HRM functions, including managing employees, refining recruitment processes, selection, evaluating candidate-job compatibility, performance management, workforce engagement and supporting practical employee training and development initiatives ( Mir, 2024; Tewari & Pant, 2020; El-Ghoul, Almassri, El-Habibi, Al-Qadi, Abou Eloun, Abu-Nasser, & Abu-Naser, 2024; Budhwar, Malik, De Silva, & Thevisuthan, 2022). This tool has a significant impact on employee engagement, streamlining repetitive administrative tasks and enabling HR professionals to concentrate on more strategic and value-adding responsibilities ( Chukwuka & Dibie, 2024; Madanchian & Taherdoost, 2025).

The global rise of AI has sparked discussions about its impact on human jobs. While some are concerned about job displacement, others argue that AI can work alongside human labour to boost productivity, innovation, and efficiency ( Spulbar & Mitrache, 2025). In the context of Human Resource Management (HRM), AI presents both opportunities and challenges (Pereira, Hadjielias, Christofi, & Vrontis, 2023). Recognising its advantages is key to encouraging its adoption ( Madanchian & Taherdoost, 2025). Table 1 below outlines the pros and cons of AI.

**Table 1.** Opportunities and challenges of AI.

Potential benefits of AI	Potential limitations of AI
Reduced costs, time, and resource expenses while maintaining or increasing personnel numbers.	The accuracy of the forecast will depend on the quality of the data obtained and the applicability of the algorithm utilised.
Increased capacity to process massive volumes of data.	Due to the “black box” nature of algorithms, it can be challenging to understand how or why an AI system made a specific prediction.
There has been an increase in the availability of previously difficult-to-obtain data, including “large quantities of resumes, social media postings, transcripts from interviews, and other forms of open-ended assessment data”.	Legal concerns are likely to arise in the future regarding the automated processing of personal data and data protection.
The possibility of improved accuracy in projecting results for outcomes that individuals and organisations deem meaningful and in which they have an interest.	AI has been affected by negative influences from members of the public, the media, and job seekers.

Source: Lourens, Vijai, Nijhawan, Garwal, Gakhreja and Saud (2024).

### AI in talent management

Hiring employees with the right skills is essential to an organisation’s success, as its overall performance is closely tied to the efforts and effectiveness of its workforce. For this reason, AI provides unique benefits by reinventing TM, with its significant potential to revolutionise how organisations attract, manage, and retain their staff ( Al-Mughairi, 2026). There is evidence in the literature that the introduction of AI is altering existing TM processes, with implications that could be negative, positive, or both, and substantial potential benefits for organisational performance ( Gao & Segumpan, 2024). AI integration in TM implies optimising several aspects of the employee lifecycle, from recruiting and selection to development, engagement, and performance tracking ( Khan, 2024; Lourens, Vijai, Nijhawan, Garwal, Gakhreja & Saud, 2024). It also has the potential to enhance personnel management by automating repetitive tasks, reducing errors, and increasing efficiency in critical areas such as recruitment, performance evaluation, employee retention, and talent acquisition ( Mir, 2024). TM is the process of placing individuals with appropriate talents and capabilities in roles that best match their strengths ( Ekhsan, Parashakti, & Perkasa, 2023). The primary goal of TM is to attract, recruit, place, develop, and retain top talent, while top talent, thus ensuring a positive vision for the future of work within an organisation ( Sowmya, Polisetty, & Dash, 2024; Briki & Gherrab, 2024).

### Guiding theoretical frameworks

Several theoretical frameworks were evaluated for their applicability to the study. The first theory that holds promise for explaining the findings is the Technology Acceptance Model (TAM) ( Venkatesh et al., 2003; Venkatesh, Thong, & Xu, 2012). TAM posits that users adopt technology more quickly when they perceive it as both helpful and easy to use. It holds promise to help explain why HR practitioners may resist using AI tools to support TM in their organisations, even though they may be aware of some of the benefits. The TAM theory was extended by the Unified Theory of Acceptance and Use of Technology (UTAUT), which added facilitating conditions, including organisational support and technical infrastructure, as well as social influence ( Venkatesh, 2012). Another theory that shows promise to make sense of AI adoption in TM is the STST (Socio-Technical Systems Theory), which emphasises that technology implementation may fail due to socio-technical misalignment ( Baxter & Sommerville, 2011). Technical issues, for example, may negatively affect employees’ trust in the technology and hinder its adoption. This holistic approach helps explain that without social interventions, such as appropriate training and change management, AI solutions are likely to fail.

## Materials and methods

### Protocol and reporting standards

A systematic literature review and a qualitative research approach were used as the research methods. A systematic review is a comprehensive literature review and analysis of results on a specific topic. Furthermore, it helps interpret findings or results. It begins with the conceptualisation of clear questions for critique by framing these questions, after which data are collected, categorised, evaluated, summarised, proofread, and discussed ( Kang & Ahn, 2018). As suggested by Molefe, Sehularoa and Koen (2024:55), five steps were followed to conduct a systematic review: 1) Conceptualisation of clear questions for critique; 2) Collection and categorisation of data; 3) Evaluation; 4) Summary and proofreading; and 5) Discussion. Searches were done between 22 April and 10 June 2025. The PRISMA 2020 rules were employed to search eight databases: Google Scholar, Scopus, Web of Science Core Collection, ScienceDirect, ProQuest, Emerald Insight, Sabinet, and El and Elsevier. Searches were conducted between 2020 and 2025.

### Quality appraisal

The researchers conducted a critical appraisal to evaluate the quality of the literature considered for eligibility. The methodological transparency, relevance, and peer review status of each publication were evaluated. Studies were weighted in the narrative synthesis rather than being disqualified on the basis of quality. Important metrics were monitored, such as regional applicability, AI domain specificity, and empirical design.

### Information source and search strategy

A comprehensive literature search was conducted across Google Scholar, Scopus, Web of Science, ScienceDirect, ProQuest, Emerald Insight, Sabinet, and Elsevier. Articles published between 2020 and 2025 in peer-reviewed journals were considered. Two independent reviewers conducted screening and data extraction. A narrative synthesis and thematic analysis were applied between April and May 2025. The search string used was: (“artificial intelligence” OR AI OR “machine learning” OR “deep learning” OR automation OR “predictive analytics”) AND (“global talent management” OR “international HR” OR “global human resource management”) AND (integration OR adoption OR implementation). The initial search yielded 1,099 results across databases, as detailed in the systematic review tracker. Duplicates were removed using Zotero’s duplicate detection tool (1087 articles remained), and articles were then exported for screening in Rayyan.

### Inclusion and Exclusion criteria

Studies were evaluated for eligibility and excluded if they did not align with the research objectives. Table 2 below shows the inclusion criteria used to choose publications for secondary data analysis.

**Table 2.** Inclusion and Exclusion criteria.

Inclusion	Exclusion
Peer-reviewed journal articles (2020–2025)	Publications before 2020
Focus on AI in the context of global talent management	Studies not focused on global talent management or lacking a central AI component
Published in English	Languages other than English
Journals listed in CABS, ABDC, or with an SJ Impact Factor	Non-peer-reviewed or grey literature
Indexed in Scopus, Web of Science, Elsevier, Sabinet, Google Scholar, ScienceDirect, Emerald Insight, or ProQuest	

Source: Researchers’ own compilation.

### Screening and selection process

*Stage 1: Title and Abstract Screening*

A PRISMA appraisal checklist and diagram were utilised to confirm that the original guidelines were followed. Two independent reviewers completed the initial screening in Rayyan to confirm that the results were relevant to the research questions. Conflicts were resolved by discussion and reassessment of the inclusion criteria. It was unnecessary to involve a third reviewer to address additional disagreements. The checklist links the PRISMA 2020 items to the pages and supplemental files in the systematic study “Integrating AI in GTM”. In this study, 1,099 articles were identified, of which 1,087 were subsequently eliminated prior to the commencement of the screening and selection process.

*Stage 2: Full-Text Screening*

A total of 272 publications were chosen for comprehensive screening, assessed according to the specified inclusion criteria:

• Concentrated specifically on the application or ramifications of AI in TM
• Examined global or transnational contexts, or inferred implications for GTM
• Published in a peer-reviewed academic journal indexed in reputable databases (e.g., Scopus, ABDC, SJIF, or CABS)
• Made theoretical, conceptual, or empirical contributions related to risk factors, performance, or acceptance of AI

**Figure 1.**
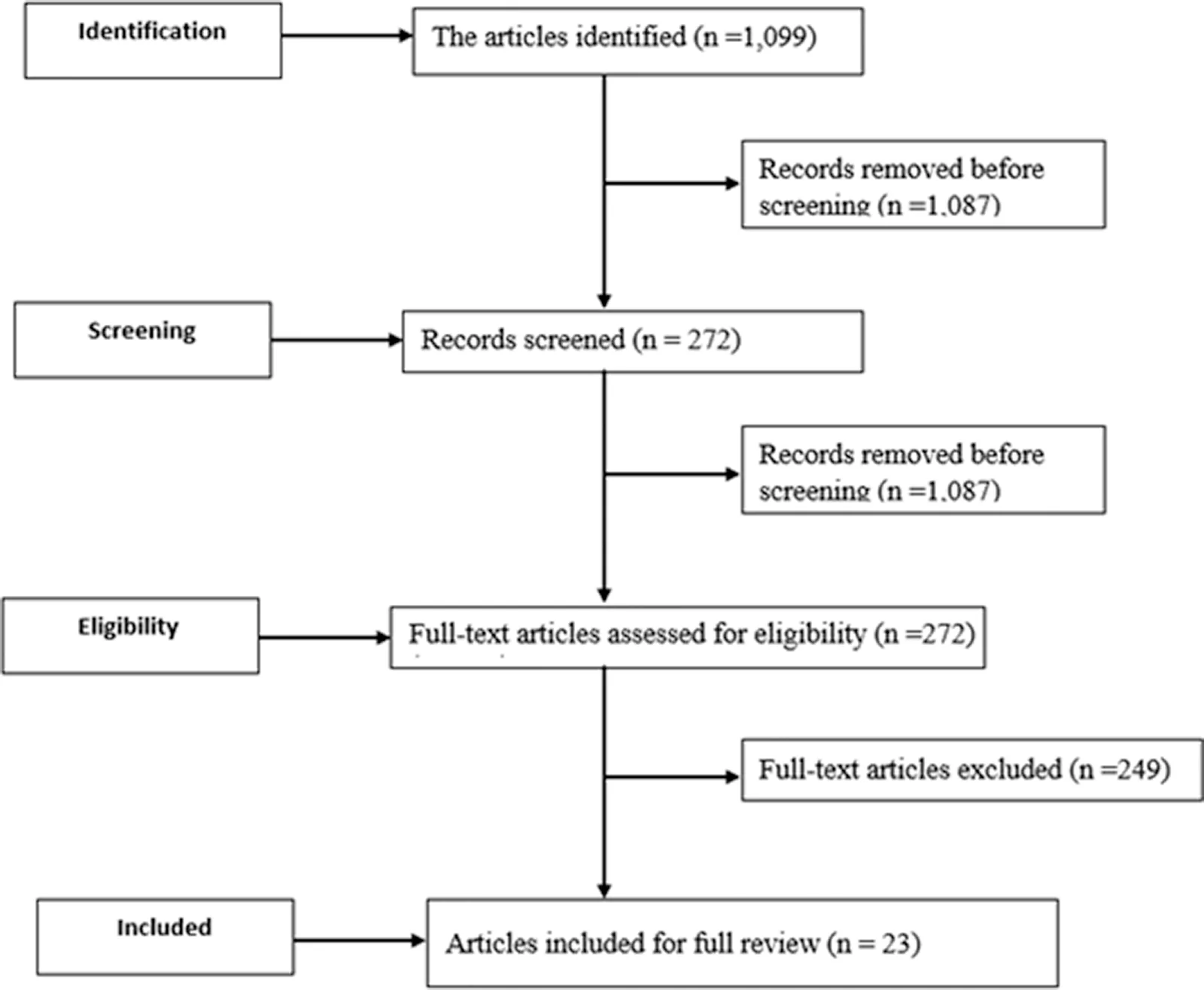
Publication selection process diagram. Source: Researchers’ own compilation.

Of 272 articles, 23 met all requirements and were selected for a comprehensive thematic synthesis. This screening process resulted in the exclusion of 249 articles. The reasons for elimination were a lack of focus on talent management, insufficient methodological transparency, and the absence of reference to AI implementation. Of these, 23 articles passed all criteria and were chosen for a comprehensive thematic synthesis. This source provides recommendations for the Preferred Reporting Items for Systematic Reviews and Meta-Analyses (PRISMA diagram) used in this review. The publication selection process diagram is presented in Figure 1 below.

### Data extraction and thematic synthesis

The main reviewer reviewed all 23 chosen articles in full, while a secondary coder checked the theme assignments for accuracy. The articles were personally annotated before being posted to Zotero and Rayyan. The following procedures were used to identify themes using thematic synthesis:

Relevant text about AI use, its facilitators and obstacles that is open-coded.

Code grouping into broad themes and sub-topics.

Linking each theme to the contributions of articles.

Thematic pattern frequency analysis.

A visual representation of the connections between themes and sub-themes.

To ensure there was no overlap and to assess conceptual clarity, the final themes were examined. The robustness of this method was increased by a comprehensive codebook, frequency table, synthesis matrix, and PRISMA-compliant visual aids.

## Presentation of findings

The study findings are discussed below.

### Geographic distribution of included studies

The systematic review’s final 23 articles cover a wide variety of geographical, sectoral, and methodological viewpoints. The findings of this study were derived from five regions. With eight publications from Asia (China, Singapore, the United Arab Emirates, and India), five from Europe (the United Kingdom, Germany, and the Netherlands), three from Africa (South Africa and Egypt), four from North America (the United States and Canada), and three from global/cross-regional studies. Asia was where most of the investigations started, then Europe.

### Methodological distribution of studies

Three methodological techniques were used in the study: conceptual, empirical, and qualitative. With eleven submissions, empirical research was the largest group, while conceptual and qualitative studies were equally represented with six each.

### Sectoral distribution of studies

The sectoral context of the collected papers was also investigated in this study. Thirteen of the 23 studies that were reviewed were conducted in the private sector, four of which looked into competitive or cross-sectoral settings, and four more of which looked into public or government organisations. With significant representation from both developed and developing economies, these distributions demonstrate a global orientation that promotes transferability and comparative insights.

### Synthesising the findings

**Figure 2.**
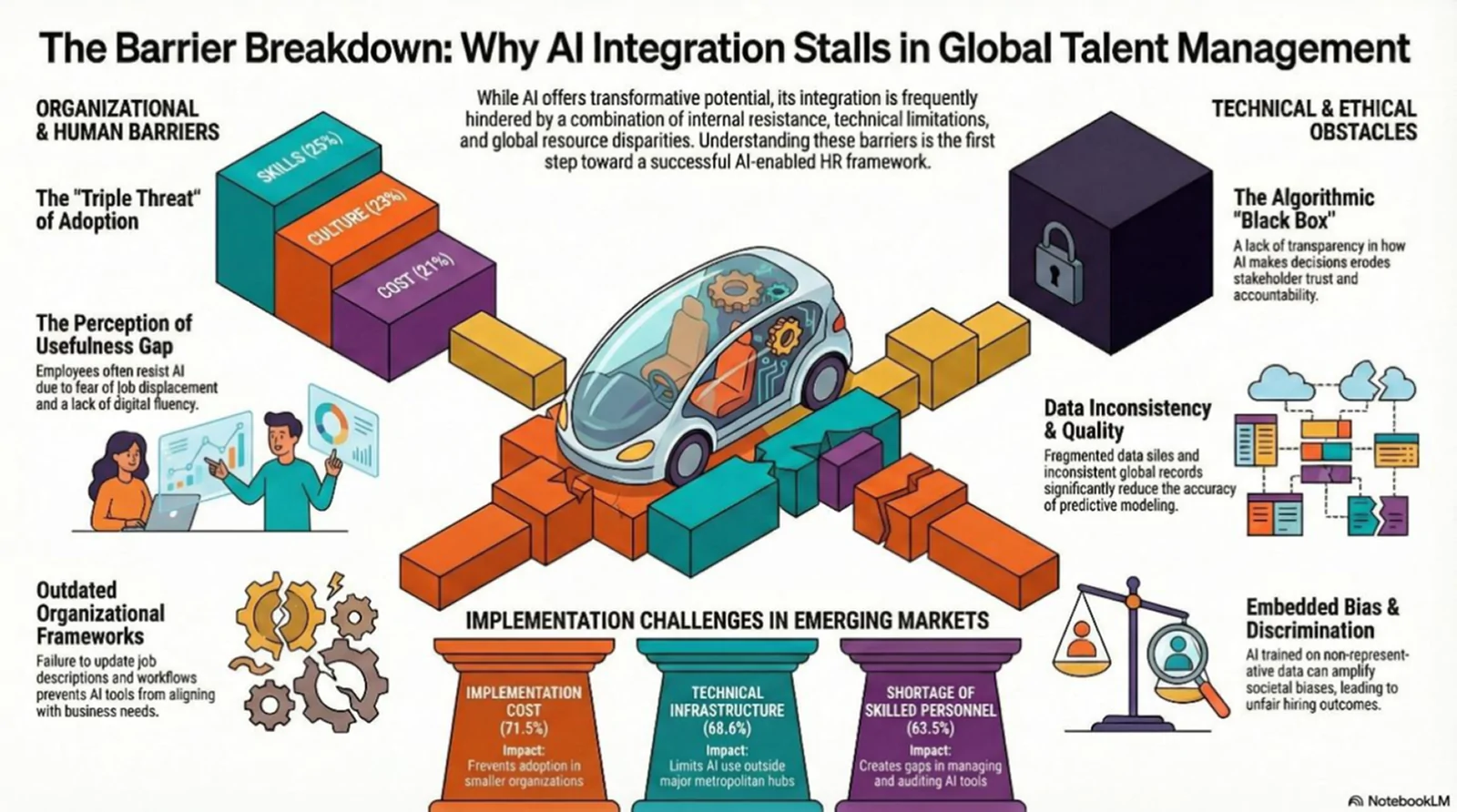
Barrier Breakdown: Why AI integration stalls in GTM. Source: Researchers’ own compilation.

The number of main themes and sub-themes identified in peer-reviewed papers supporting the incorporation of AI into GTM is summarised in Table 3. The PRISMA diagram above shows that themes and sub-themes were developed from 23 articles. While AI offers considerable promise, its implementation in global talent GTM is shaped by governance and accountability, implementation barriers, and ethical and technical risks. The primary themes and subthemes are summarised in Table 4, along with definitions and examples of each theme and subtheme. Additionally, Figure 2 illustrates why AI integration stalls in GTM.

**Table 3.** Theme/Author distribution: AI in Global Talent Management.

Research question	Theme	Sub-theme	Supporting articles (APA Format)
What are the primary barriers and risks associated with AI implementation in GTM?	Ethical Governance and Accountability	Algorithmic Bias and Decision Opacity	Varma et al. (2024); Roppelt et al. (2025); Sulaiman et al. (2025)
		Worker Dignity and Dehumanisation	Varma et al. (2024); Alam et al. (2025); Roppelt et al. (2025)
	Contextual and Implementation Barriers	Challenges in Emerging Markets	Sulaiman et al. (2025); Thanvi et al. (2024)
		Resistance and Anxiety	Smith et al. (2025); Roppelt et al. (2025); Varma et al. (2024)
	Ethical and Technical Risks	Data Quality and Integrity	Kingsley (2025); Smith et al. (2025); Sulaiman et al. (2025)
		Security and Post-Adoption Harm	Roppelt et al. (2025); Varma et al. (2024); Kingsley (2025)

Source: Researchers’ own compilation.

**Table 4.** Summary of themes and sub-themes.

Main theme	Sub theme	Definition	Example indicators
Governance and Accountability	Bias & Opacity	Ethical concerns over the lack of transparency and the potential for AI systems to reinforce inequality.	Undisclosed screening algorithms, fairness audits, and algorithmic clarification gaps
	Stakeholder Trust	Trustworthiness of AI use from the perspective of job candidates, employees, and decision-makers.	Perceived fairness of AI decisions, candidate feedback, and opt-out options
Implementation Barriers	Resistance & Fear	Pushback from employees or managers due to fear of surveillance, job loss, or unfamiliarity.	Negative perceptions of automation, reluctance to engage with AI, opposition to rollout
	Skills & Integration Gaps	A lack of technical skills or system compatibility is needed to support AI deployment.	Insufficient digital maturity, low AI literacy, and siloed legacy systems
Ethical & Technical Risks	Data Quality	Issues related to data completeness, bias, and labelling that impact AI model reliability.	Inconsistent labelling, outdated records, and biased training data
	Over-Automation	Use of AI in emotionally sensitive or discretionary decisions without adequate human oversight.	AI-led terminations, fully automated hiring, lack of human-in-the-loop controls

Source: Researchers’ own compilation.

## Discussion

**Figure 3.**
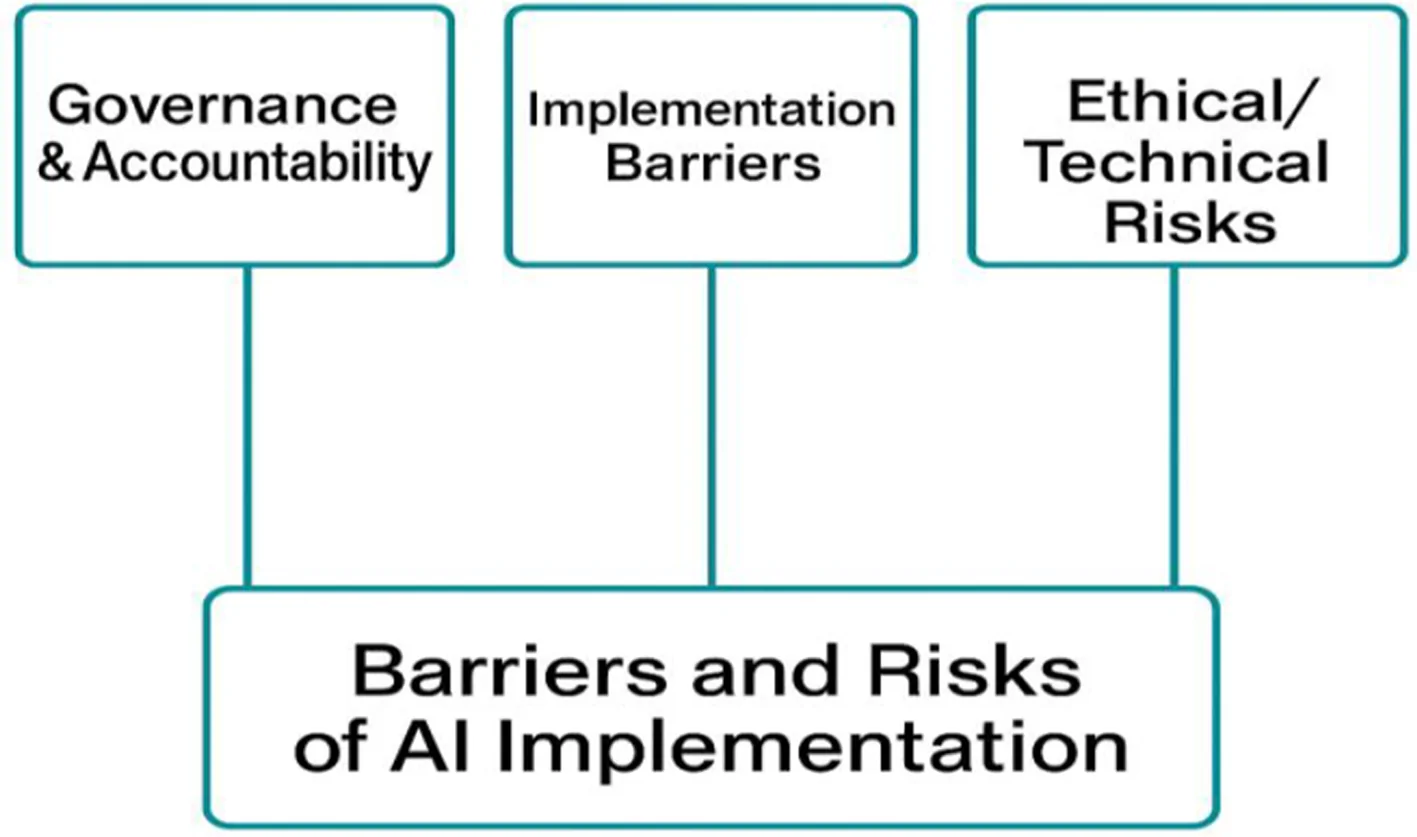
Barriers and risks of AI implementation. Source: Researchers’ own compilation.

The primary purpose of this study was to explore barriers and risks that hinder this integration. This study seeks to answer this research question: What are the primary barriers and risks associated with AI implementation in GTM? While AI offers considerable promise, its implementation in GTM is fraught with challenges. Three overarching themes emerged from the literature: governance and accountability, implementation barriers, and ethical and technical risks. Figure 3 presents a visualisation of the main Barriers and risks of AI implementation in GTM.

The application of AI in HRM processes, including GTM, has huge potential to revolutionise processes and improve organisational efficiency ( Madanchian & Taherdoost, 2025). Even if the field of human resource management will benefit from the deep integration of AI and talent management in the future, HR professionals must be aware of the risks and potential obstacles associated with AI deployment in GTM ( Lutfi & Mohammadi, 2025). In this study, issues such as governance and accountability, implementation barriers and ethical and technical risks were identified as the main obstacles to AI implementation in GTM.

### Theme 1: Governance and Accountability

This theme encompasses the ethical, legal, and procedural frameworks needed to ensure AI use in HR is fair, transparent, and accountable.

• *Sub-theme: Algorithmic Bias and Decision Opacity*

The risk is that “black box” algorithms lack transparency and may replicate historical inequities in hiring and promotion ( Varma et al., 2024; Roppelt et al., 2025). Organisations must audit algorithms and introduce fairness controls, or risk eroding gains in inclusion ( Sulaiman et al., 2025). Organisations must audit algorithms and introduce fairness controls or risk eroding equity and inclusion gains in GTM.

• *Sub-theme: Stakeholder Trust and Transparency*

The ethical concern is that over-reliance on algorithms treats humans as objects to be acted upon (“patiency”) rather than autonomous agents ( Varma et al., 2024). There is also a risk of dehumanisation in processes requiring high empathy, such as candidate rejection ( Varma et al., 2024). Without explainable outputs, trust declines, especially in rejection decisions or promotion denials. Transparent communication about how AI influences HR decisions must be embedded in practice. Ethical lapses or a lack of transparency can erode trust and legitimacy. Governance structures must evolve in tandem with AI innovation to ensure fairness and regulatory compliance.

### Theme 2: Implementation Barriers

This theme captures the organisational, cultural, and environmental obstacles, such as resistance to change and infrastructure deficits, that hinder the smooth adoption and integration of AI systems across global and local contexts.

• *Sub-theme: Challenges in Emerging Markets*

Regional factors, including infrastructure limitations, language processing gaps, and traditional hiring preferences, hinder the implementation of standard AI models. In South Asian corporate sectors, particularly in Pakistan, implementation is significantly hindered by a cultural preference for personal connections in hiring, which 76.6% of respondents identified as a significant factor ( Sulaiman et al., 2025). This relationship-oriented business culture, where referrals and personal networks traditionally dominate, creates tension with standardised, algorithm-based selection tools ( Sulaiman et al., 2025; Hussain et al., 2022). Furthermore, hierarchical decision-making structures emerge as a major cultural barrier, affecting how AI recommendations are integrated into final selection processes ( Sulaiman et al., 2025). Technical infrastructure limitations, such as internet connectivity issues and frequent power outages, represent practical barriers for 76% of organisations in these regions ( Sulaiman et al., 2025). Language processing gaps also pose a critical technical barrier, as 60% of senior managers report that current AI systems struggle to accurately interpret bilingual resumes that mix English with regional languages such as Urdu or culturally specific terminology ( Sulaiman et al., 2025). Finally, 84% of respondents cite data quality issues arising from inconsistent standards in regional education and non-standardised employment verification records, which complicates algorithm training and validation ( Sulaiman et al., 2025).

• *Sub-theme: Resistance and Anxiety*

The fear of job loss, loss of autonomy, and workplace surveillance among employees leads to reduced engagement or deliberate tool avoidance. A primary driver of resistance is “automation anxiety,” particularly among “white-collar” workers who fear that AI will eventually replace them for cost-efficiency reasons ( Smith et al., 2025; Roppelt et al., 2025). Public sector organisations, such as the UK Civil Service, are often perceived as risk-averse and slow to respond to change, which further inhibits the adoption of transformative technologies despite staff’s personal readiness ( Smith et al., 2025). Within the management layer, a generational gap in trust exists where 68% of senior leaders raised in traditional business environments remain sceptical of algorithmic decisions, preferring to rely on their own “gut feeling” and experience ( Sulaiman et al., 2025).

Additionally, employees may perceive AI as intrusive, which can diminish trust and reduce engagement if transparency regarding the system’s purpose is lacking ( Smith et al., 2025; Varma et al., 2024). This resistance is often compounded by an attitudinal “deskilling” in which workers feel treated as objects to be acted upon (“patiency”) rather than as autonomous agents, potentially leading to the atrophy of professional skills ( Varma et al., 2024). Successful implementation, therefore, requires addressing organisational change management and targeted upskilling rather than just technical deployment ( Smith et al., 2025; Roppelt et al., 2025).

### Theme 3: Ethical and Technical Risks

This theme captures the inherent risks and moral dilemmas associated with applying machine learning and algorithmic logic to sensitive human domains, encompassing both technical failures in systems and the erosion of ethical standards in people management.

• *Sub-theme: Data Quality and Integrity*

The “garbage in, garbage out” (GIGO) syndrome, where inconsistent data standards, missing fields, or poor-quality datasets erode the reliability, validity, and accuracy of AI-driven predictive models. A critical technical barrier to effective AI integration is the reliance on raw data that may be fragmented or inaccurate. The phenomenon of “garbage in, garbage out” remains a pervasive concern; if automated processes utilise erroneous inputs, they will efficiently reach entirely incorrect conclusions regarding candidate suitability or workforce forecasts ( Kingsley, 2025; Smith et al., 2025). Organisations often face challenges with inconsistent labelling and siloed systems that hinder model accuracy ( Kingsley, 2025; Roppelt et al., 2025). In emerging markets like Pakistan, this is exacerbated by a lack of standardised documentation, such as non-standardised experience letters or bilingual resumes that current systems struggle to interpret accurately ( Sulaiman et al., 2025). Furthermore, when predictive algorithms are trained on small or non-representative datasets, they risk producing results that are both inaccurate and inherently biased ( Roppelt et al., 2025).

• *Sub-theme: Security and Post-Adoption Harm*

The emergence of negative consequences and systemic vulnerabilities following adoption, including cybersecurity threats, privacy violations, and the potential for technological tools to dehumanise human resource processes. Beyond initial implementation, organisations must navigate “Harmful Forms of Practice” (HFP) that can manifest as data privacy breaches, information leaks to third parties, and system downtimes ( Roppelt et al., 2025). Fears regarding unauthorised access to sensitive performance data and the potential for “surveillance-like” analytics often erode stakeholder trust and adversely affect adoption intentions ( Kingsley, 2025; Roppelt et al., 2025). Beyond technical security, there is a profound risk of dehumanisation within the HR function. Over-reliance on predictive algorithms can subordinate worker dignity, treating humans as objects to be acted upon (“patiency”) rather than autonomous agents (“agency”) ( Varma et al., 2024). This is particularly critical in processes requiring high empathy, such as candidate rejection or professional development coaching; replacing human discretion with “black box” algorithms risks alienating users and undermining the core human-centric values of talent management ( Varma et al., 2024; Aguinis et al., 2024). Therefore, human-in-the-loop systems remain essential to mitigate the risk of treating human capital as a mere commodity ( Varma et al., 2024; Roppelt et al., 2025).

## Conclusion

The study sought to explore barriers and risks that hinder AI integration into GTM. The findings identified three key barriers: governance and accountability, implementation barriers, and ethical and technical risks. These factors significantly hinder the successful adoption of AI within GTM. For organisations to fully benefit from AI integration, it is essential that these barriers are effectively addressed. This study provides HR practices with insightful evidence to focus on factors that may hinder the successful implementation of AI, particularly in GTM. In practical terms, this study will help HR practitioners to establish clear guidelines to promote the responsible and effective use of AI within HR departments, ensuring accountability among employees. HR practitioners globally should prioritise equipping staff with the necessary skills to foster productive collaboration between human workers and AI systems. Ultimately, to foster trust and confidence in AI adoption, management must establish and maintain fair, transparent processes across the organisation. This study presents several limitations and suggests avenues for future research. Firstly, it relies on secondary rather than primary data sources. Secondly, the data were drawn from eight databases, namely Google Scholar, Scopus, Web of Science, ScienceDirect, ProQuest, Emerald Insight, Sabinet, and Elsevier, which may have led to the exclusion of certain relevant information. Thirdly, the analysis was limited to publications from 2020 to 2025, potentially omitting significant studies published prior to 2020. Lastly, only articles published in English were considered, potentially excluding valuable insights from non-English publications.

## Data Availability

The checklist and flowchart supporting this study are available in Zenodo at: https://doi.org/10.5281/zenodo.19651598 ( Maake, Bezuidenhout, Schultz & Smit, 2026). Data are available under the terms of the Creative Commons Attribution 4.0 International license (CC-BY 4.0).

## References

[ref1] Aguinis H Beltran JR Cope A : How to use generative AI as a human resource management assistant. *Organ. Dyn.* 2024;53(1):101029–101027. 10.1016/j.orgdyn.2024.101029

[ref75] Al-Mughairi AM : *Artificial Intelligence (AI) into talent management. In Aligning talent management and organizational innovation goals.* IGI Global Scientific Publishing.2026;107–138.

[ref2] Alam R Lantara N Razak AR : The role of artificial intelligence in recruitment: Examining candidate experience as a mediator and organizational culture as a moderator in quality of hires. *Asian Management and Business Review.* 2025;5(1):160–177.

[ref3] Alawamleh M Shammas N Alawamleh K : Examining the limitations of AI in business and the need for human insights using Interpretive Structural Modelling. *J. Open Innov.: Technol. Mark. Complex.* 2024;10(3):100338–100317. 10.1016/j.joitmc.2024.100338

[ref4] Amushila J Bussin MH : The effect of talent management practices on employee retention at the Namibia University of Science and Technology: Administration middle-level staff. *SA J. Hum. Resour. Manag.* 2021;19:1–11. 10.4102/sajhrm.v19i0.1485

[ref5] Babashahi L Barbosa CE Lima Y : AI in the workplace: A systematic review of skill transformation in the industry. *Administrative Sciences.* 2024;14(6):1–28. 10.3390/admsci14060127

[ref6] Baxter G Sommerville I : Socio-technical systems: From design methods to systems engineering. *Interact. Comput.* 2011;23(1):4–17. 10.1016/j.intcom.2010.07.003

[ref7] Benhamou S : Artificial intelligence and the future of work. *Rev. Econ. Ind.* 2020;169:57–88. 10.4000/rei.8727

[ref9] Bhardwaj G Singh SV Kumar V : An empirical study of artificial intelligence and its impact on human resource functions. *2020 International Conference on Computation, Automation and Knowledge Management.* 2020;47–51.

[ref11] Booyse D Scheepers CB : Barriers to adopting automated organisational decision-making through the use of artificial intelligence. *Manag. Res. Rev.* 2024;47(1):64–85. 10.1108/MRR-09-2021-0701

[ref12] Boštjančič E Slana Z : The role of talent management comparing medium-sized and large companies–major challenges in attracting and retaining talented employees. *Front. Psychol.* 2018;9:1–10. 10.3389/fpsyg.2018.01750 30283391 PMC6156250

[ref78] Briki M Gherrab S : Leveraging Artificial Intelligence to optimize talent management in Higher Education Institutions. *ATRAS journal.* 2024;5(3):464–480. 10.70091/atras/AI.29

[ref14] Budhwar P Malik A De Silva MT : Artificial intelligence–challenges and opportunities for international HRM: a review and research agenda. *Int. J. Hum. Resour. Manag.* 2022;33(6):1065–1097. 10.1080/09585192.2022.2035161

[ref15] Bujold A Roberge-Maltais I Parent-Rocheleau X : Responsible artificial intelligence in human resources management: a review of the empirical literature. *AI and Ethics.* 2024;4(4):1185–1200. 10.1007/s43681-023-00325-1

[ref17] Chukwuka EJ Dibie KE : Strategic role of artificial intelligence (AI) on human resource management (HR) employee performance evaluation function. *International Journal of Entrepreneurship and Business Innovation.* 2024;7(2):269–282. 10.52589/IJEBI-HET5STYK

[ref72] Diyin S Bhaumik B Wang D : The influence of Artificial Intelligence on talent acquisition and human resource management. *J. Electrical Systems.* 2024;20(11):4868–4878. 10.52783/jes.8824

[ref84] El-Ghoul M Almassri MM El-Habibi MF : AI in HRM: Revolutionizing recruitment, performance management, and employee engagement. *International Journal of Academic and Applied Research.* 2024;8(9):16–23.

[ref85] Ekhsan M Parashakti RD Perkasa DH : The impact of talent management on employee performance mediated by employee engagement. *East Asian Journal of Multidisciplinary Research.* 2023;2(4):1821–1834. 10.55927/eajmr.v2i4.3913

[ref19] Faqihi A Miah SJ : Artificial intelligence-driven talent management system: Exploring the risks and options for constructing a theoretical foundation. *Journal of Risk and Financial Management.* 2023;16(1):1–18. 10.3390/jrfm16010031

[ref20] Farouk K : Artificial intelligence and the future of work: implications for workers and businesses. *Inksta publishers.* 2025;134–140.

[ref21] Gallardo-Gallardo E Thunnissen M Scullion H : Talent management: context matters. *Int. J. Hum. Resour. Manag.* 2020;31(4):457–473. 10.1080/09585192.2019.1642645

[ref22] Gao S Segumpan RG : The effect of AI-driven talent management on organizational performance among retail SMEs: A systematic review. *IBIMA Business Review.* 2024;1–9. 10.5171/2024.588377

[ref23] Ghaleche SA : Assessing Employee Perceptions of AI Integration in Workplace Decisions. *Journal of Resource Management and Decision Engineering.* 2023;2(4):24–30.

[ref24] Ghosh R Nachmias S Murdoch D : Prioritizing humans: HRD’s vital role in AI adoption for workplace success. *Hum. Resour. Dev. Int.* 2024;27(3):319–323.

[ref27] Hussain Z Mari IH Rind AA : Exploring the barriers of Artificial Intelligence Adoption in Digital Marketing Landscape. *ULIL ALBAB: Jurnal Ilmiah Multidisiplin.* 2022;1(4):824–835.

[ref77] Kang H Ahn E : Introduction to systematic review and meta-analysis. *Korean Journal of Anesthesiology.* 2018;71(2):103–112. 10.4097/kjae.2013.64.2.103 29619782 PMC5903119

[ref30] Khan MR : Application of artificial intelligence for talent management: Challenges and opportunities. *Intelligent human systems integration (IHSI 2024): Integrating people and intelligent systems.* 2024;119(119):4–7.

[ref31] Kingsley J : From data lakes to talent pipelines: leveraging big data and AI for workforce forecasting. 2025.

[ref74] Liu S Li G Xia H : Analysis of talent management in the artificial intelligence era. *In the 5th Asia-Pacific Conference on Economic Research and Management Innovation.* 2021:38–42.

[ref71] Lourens M Vijai C Nijhawan G : Artificial Intelligence-Driven Talent Management System: Exploring the Risks and Options Sustainable Success. *In 2024 4th International Conference on Innovative Practices in Technology and Management (ICIPTM).* 2024; pp.1–6.

[ref34] Lutfi L Mohammadi A : Challenges of Implementation Artificial Intelligence in Human Resources Management. *International Journal of Academic Research in Business and Social Sciences.* 2025;15(4):1–12. 10.6007/IJARBSS/v15-i4/24796

[ref35] Maake G Bezuidenhout A Schultz CM : Checklist and Flowchart for Explore barriers and risks that hinder AI integration into global talent management: A Systematic Review. *Zenodo.* 2026. 10.5281/zenodo.19651598

[ref36] Madanchian M Taherdoost H : Barriers and enablers of AI adoption in human resource management: A critical analysis of organizational and technological factors. *Inform.* 2025;16(1):1–16. 10.3390/info16010051

[ref41] Mir AI : Application of AI in talent management: a systematic review of benefits, challenges, and prospects. *Academy of Education and Social Sciences Review.* 2024;4(4):612–630. 10.48112/aessr.v4i4.941

[ref83] Molefe LL Sehularoa L Koen D : Support needed by workers providing care to children with cognitive disabilities: A systematic review. *Journal for New Generation Science.* 2024;22(1):52–67. 10.47588/jngs.2024.22.01.a4

[ref42] Murire OT : Artificial Intelligence and Its Role in Shaping Organisational Work Practices and Culture. *Administrative Sciences.* 2024;14(12):1–16.

[ref43] Mwita KM Kitole FA : Potential benefits and challenges of artificial intelligence in human resource management in public institutions. *Discover Global Society.* 2025;3(1):35. 10.1007/s44282-025-00175-8

[ref45] Ochieng EM : A Study of the History Functions Roles and Challenges of Human Resources Management. *Journal of Enterprise and Business Intelligence.* 2023;3(1):54–064. 10.53759/5181/JEBI202303006

[ref47] Pandey S Khaskel P : Application of AI in human resource management and gen Y’s reaction. *Int. J. Recent Technol. Eng.* 2019;8(4):10325–10331. 10.35940/ijrte.D4585.118419

[ref48] Poisat P Cullen M Calitz AP : Human resource managers' perceptions on the impact of Al on the South African workforce. *SA J. Hum. Resour. Manag.* 2024;22:1–13.

[ref50] Roppelt JS Schuster A Greimel NS : Towards effective adoption of artificial intelligence in talent acquisition: A mixed-method study. *Int. J. Inf. Manag.* 2025;82:102870–102818. 10.1016/j.ijinfomgt.2025.102870

[ref51] Rosário C : Benefits and risks of using AI in Human Resource Management: Literature review. *Educational Research (IJMCER).* 2025;7(2):146–154.

[ref81] Rožman M Oreški D Tominc P : Integrating artificial intelligence into a talent management model to increase the work engagement and performance of enterprises. *Frontiers in psychology.* 2022;13:1–15. 10.3389/fpsyg.2022.1014434 PMC9732559 36506984

[ref52] Sareen P Mishra S : A study of talent management and its impact on performance of organizations. *J. Bus. Manag.* 2016;18(12):66–73.

[ref53] Scheffknecht T : *AI in workplace: Exploring key factors for employee adoption and integration.* FH Vorarlberg Fachhochschule Vorarlberg;2025. Doctoral dissertation.

[ref80] Sindhura K : Talent management strategies in human resource management: critical for business-a systematic review. *Journal of Positive School Psychology.* 2022;6(3):3396–3409.

[ref55] Singh A Pandey J : Artificial intelligence adoption in extended HR ecosystems: enablers and barriers. An abductive case research. *Front. Psychol.* 2024;14:1–13. 10.3389/fpsyg.2023.1339782 PMC10847531 38327504

[ref56] Smith M Prabhakar G Nisar TM : Adoption of AI by the HR function in the civil service. *Employee Relations: The International Journal.* 2025;47(1):104–126. 10.1108/ER-02-2024-0096

[ref73] Sowmya G Polisetty A Dash G : Leveraging Artificial Intelligence for Talent Management. *In Handbook of Artificial Intelligence Applications for Industrial Sustainability.* 2024:124–143. 10.1201/9781003348351-9

[ref57] Spulbar LF Mitrache LA : How humans and AI can thrive together in the workplace? Revista de Științe Politice. *Revue des Sciences Politiques.* 2025;86:274–287.

[ref58] Sulaiman G Fuzail M Urooge S : The role of artificial intelligence in hr: a study of the impact of AI-powered recruitment tools on hiring decisions. *Center for Management Science Research.* 2025;3(3):10–30.

[ref60] Tewari I Pant M : Artificial intelligence reshaping human resource management: A review. *2020 IEEE International Conference on Advent Trends in Multidisciplinary Research and Innovation.* 2020;1–4.

[ref61] Thanvi AUH Zafar H Siddiqui FA : HR Professionals’ Intention to Adopt and Use of Artificial Intelligence in Recruiting Talents in Retail Industry of Karachi (Pakistan). *Research Journal for Social Affairs.* 2024;2(4):223–244. 10.71317/RJSA.002.04.0191

[ref62] Tiwari R Babu NS Marda K : The impact of artificial intelligence in the workplace and its effect on the digital wellbeing of employees. *International Journal of Progressive Research in Engineering Management And Science.* 2024;4(6):2422–2427.

[ref63] Tsaousiotis K Panitsidis K Spinthiropoulos K : A New Perspective on Talent Management: An Integrative Review of the Current Literature. *Administrative Sciences.* 2025;15(3):1–16. 10.3390/admsci15030102

[ref65] Varma NK Rajeswari MS : A study on talent management in Venkata Narayana active ingredients. *International Journal of Research in Engineering, IT and Social Sciences.* 2024;14(7):127–142.

[ref66] Venkatesh V Morris MG Davis GB : User acceptance of information technology: Toward a unified view. *MIS Q.* 2003;27(3):425–478. 10.2307/30036540

[ref67] Venkatesh V Thong JY Xu X : Consumer acceptance and use of information technology: Extending the unified theory of acceptance and use of technology. *MIS Q.* 2012;36(1):157–178. 10.2307/41410412

[ref79] Vrontis D Christofi M Pereira V : Artificial intelligence, robotics, advanced technologies and human resource management: a systematic review. *Artificial intelligence and international HRM.* 2023:172–201. 10.4324/9781003377085-7

[ref69] Zake GB Jonck P Pelser AM : Impact of contextual factors on organisational performance mediated by talent management. *SA J. Ind. Psychol.* 2024;50(1):1–12. 10.4102/sajip.v50i0.2184

[ref70] Zavodna LS Überwimmer M Frankus E : Barriers to the implementation of artificial intelligence in small and medium-sized enterprises: Pilot study. *Journal of Economics and Management.* 2024;46:331–352. 10.22367/jem.2024.46.13

