# Supplementary material for: Explore barriers and risks that hinder AI integration into global talent management: A Systematic Review
Source: F1000Res. 2026 May 25;15:791. [Version 1] doi: 10.12688/f1000research.179429.1 (PMC13554726; doi:10.12688/f1000research.179429.1)
Supplement: Peer review history

# Reviewer report: Reviewer response for version 1

**DOI:** 10.5256/f1000research.197938.r490343

**Published:** 2026-07-15

**Authors:** Packiyanathan Mathushan

**Affiliations:** 1 University of Vavuniya, Vavuniya, Sri Lanka

01. Change the topic – "Exploring Barriers and Risks Hindering AI Integration into Global Talent Management: A Systematic Review.”

 02. The abstract says the review followed **PRISMA-ScR**, but the method section refers to **PRISMA 2020**. These are not the same. The authors must clearly state whether it is a systematic review or a scoping review.

 03. The article says **1,099 articles were identified**, **1,087 remained after duplicates**, but later says **1,087 were eliminated before screening** and **272 were screened**. These numbers do not logically match.

 04. The search string focuses mainly on “global talent management” and “international HR,” which may miss relevant studies using terms such as *e-HRM, digital HRM, AI recruitment, algorithmic HRM, workforce analytics,* or *people analytics*.

 05. TAM, UTAUT, and Socio-Technical Systems Theory are mentioned, but they are not strongly integrated into the findings or discussion. The theory section feels added rather than analytically used.

 06. The review identifies themes but does not deeply compare studies, explain contradictions, or show which barriers are most influential across regions, sectors, or methodologies.

Are the rationale for, and objectives of, the Systematic Review clearly stated?

Yes

Is the statistical analysis and its interpretation appropriate?

Partly

If this is a Living Systematic Review, is the ‘living’ method appropriate and is the search schedule clearly defined and justified? (‘Living Systematic Review’ or a variation of this term should be included in the title.)

Yes

Are sufficient details of the methods and analysis provided to allow replication by others?

Partly

Are the conclusions drawn adequately supported by the results presented in the review?

Yes

Reviewer Expertise:

HRM, Entrepreneurship, Management, Strategic management, organisational innovation

I confirm that I have read this submission and believe that I have an appropriate level of expertise to confirm that it is of an acceptable scientific standard.

# Reviewer report: Reviewer response for version 1

**DOI:** 10.5256/f1000research.197938.r488505

**Published:** 2026-07-15

**Authors:** Fatima Touhami

**Affiliations:** 1 Université Sultan Moulay Slimane, Béni-Mellal, Morocco

**Reviewer Report**

 This systematic review addresses a timely and relevant topic by examining the barriers and risks associated with implementing Artificial Intelligence in Global Talent Management. The review objective is clearly stated, and the study follows a structured systematic review approach based on PRISMA guidelines. The findings are well organized and provide useful insights into governance, implementation, and ethical challenges.

 However, several methodological aspects require clarification, particularly regarding the quality appraisal process, search strategy reproducibility, and justification for excluding a large number of studies during screening. In addition, the discussion could be strengthened by providing a more critical analysis of the included studies and their methodological limitations.

 Overall, the manuscript makes a valuable contribution to the literature and offers practical implications for HR professionals. I recommend **acceptance with minor revisions**.

Are the rationale for, and objectives of, the Systematic Review clearly stated?

Partly

Is the statistical analysis and its interpretation appropriate?

No

If this is a Living Systematic Review, is the ‘living’ method appropriate and is the search schedule clearly defined and justified? (‘Living Systematic Review’ or a variation of this term should be included in the title.)

No

Are sufficient details of the methods and analysis provided to allow replication by others?

Yes

Are the conclusions drawn adequately supported by the results presented in the review?

Partly

Reviewer Expertise:

Marketing - Human Ressources - Economy - Management

I confirm that I have read this submission and believe that I have an appropriate level of expertise to confirm that it is of an acceptable scientific standard.

# Reviewer report: Reviewer response for version 1

**DOI:** 10.5256/f1000research.197938.r498037

**Published:** 2026-07-08

**Authors:** Paul Boselie

**Affiliations:** 1 Utrecht University, Utrecht, The Netherlands

Explore barriers and risks that hinder AI integration into global talent management:

 A Systematic Review

 July, 2026

 This paper combines two relevant and contemporary themes in human resource management (HRM): talent management and AI in employment relationships. Talent management (TM) is a hot topic within the broader academic HRM discipline (since 2010). There are multiple literature reviews on TM, but this one is linked to increased digitalization through artificial intelligence (AI).

 INTRODUCTION

 The first sentence of the paper states “the work environment is rapidly changing due to the constantly rising global disruptions.” What kind of disruptions? And how does this relate to the grand challenges such as increased digitalization, growing inequality, aging populations etc.? It is not clear how these disruptions or challenges relate to talent management.

 The second paragraph is somewhat confusing, partly because it introduces different concepts – talent management (TM), human resource management (HRM) and human resource development (HRD) – without explaining how these rather broad concepts relate to each other. I suggest at least removing HRD and focus on TM and HRM with TM as part of HRM, just like performance management (PM) can be seen as part of HRM.

 It is not clear how TM is defined here. See for example the work of Marian Thunnissen who makes a distinction between inclusive and exclusive TM, but also between object and subject approaches. See her overviews in IJHRM and HRMR from 2013 or some of her other scientific publications. AI skills can be linked to inclusive TM and object approaches in TM given (up)skilling requirements due to further digitalization.

 PROBLEM STATEMENT

 It is not clear how AI is defined here. See for example the work of Budwhar et al. (2023) in HRMJ. To what extent is the focus for example on large language models (LLM)? AI can be much broader including advanced techniques and algorithms that can also be used in HRM, for example in selective recruitment and selection.

 To clarify your problem statement you need to define and specify both TM and AI. In addition you need to clarify and specify the scope of AI and TM/HRM, for example in terms of:

- AI implications for HR professionals;
- AI professionals such a IT experts;
- AI implications for HRM policies, HRM practices and work design;
- AI implications for employees;
- Etc.

Do not try to boil the ocean in one paper. You need focus.

 Why focus on global talent management (GTM)? Explain and clarify. You might just want to focus on talent management in general. GTM relates to International HRM and might suggest some kind of expatriate approach in relationship to AI developments. Is that what your paper is about? Again, try to focus and minimize the number of different concepts such as TM, HRM, GTM, HRD etc.

 LITERATURE REVIEW

 What is different in the AI development in comparison to other known technological developments? Here are some suggestions:

- AI has a potential impact on certain services functions such as secretaries and accountants;
- AI has an impact on knowledge workers, in particular those working in services. That also includes professionals such as lawyers.

Table 1 on opportunities and challenges of AI can be strengthened an elaborated by a threefold distinction between (1) positive effects, (2) negative effects and (3) potential risks. Potential risks can include issues such as ethics and privacy. The way organizations deal with these potential risks can have positive or negative effects.

 AI in talent management

 Here it is not clear what a talent is? Again the inclusive-exclusive and object-subject TM approaches can be helpful. In a way AI can be disruptive. The A-players in the past can become the vulnerable workers of the near future, because AI is taking over there uniqueness and strategic value for the organization. Think of the accountant and the laywer.

 A big theme in AI and employment relationships is the need for upskilling. And linked to upskilling is another hot topic: job polarization. This latter refers to the potential gaps between employee groups who can make the move towards new jobs (tasks including AI) and those who are not able to develop towards new jobs and tasks. Job polarization in the past was very much linked to higher and lower educated workers. With the rapid AI development job polarization could also emerge within higher education workers, in particular those in services functions. The electricians and plumbers are probably less vulnerable due to AI developments.

 Guiding theoretical frameworks

 The theory of Technology Acceptance Model (TAM) sounds interesting and relevant. There are two other theoretical frameworks mentioned in the section (UTAUT and STST), however, the theories are not fully explained. This section needs to be elaborated and should lead to some kind of set of expectations, propositions or hypotheses.

 What is the conceptual model of the paper? And would it be possible to visualize the conceptual model including the key concepts of the study?

 MATERIALS AND METHODS

 What is the logic behind the choice for choosing 2020-2025? AI developments mainly emerged in 2023, for example ChatGPT.

 In addition it is not clear how the search was performed in the databases mentioned. Collecting articles for a systematic review is a lot of work and causes a lot of discussions on the inclusion and exclusion of papers or articles. You need to provide more information on the content criteria for selection (See Figure 1). The Figure mainly provides an overview of the process and procedure, not so much on the content criteria. This also relates to issues of definitions of the key concept of TM and AI. See comments above.

 23 articles for further analyses is limited to some extent.

 PRESENTATION OF THE FINDINGS

 The findings are rather descriptive: countries, sectors and general themes. Can you be more specific here, also including more information on the definitions of the key concepts of TM and AI?

 DISCUSSION

 First sentence of this paragraph says “The primary purpose of this study was to explore barriers and risks that hinder this integration.” I am not fully convinced that the approach and the results meet this purpose.

 “While AI offers considerable promise, its implementation in GTM is fraught with challenges. Three overarching themes emerged from the literature: governance and accountability, implementation barriers, and ethical and technical risks.” You need to be very careful with this statement, because this is what the literature presents in the 23 articles that were studied in the paper. This is what science presents in publications, but what about TM and AI in practice? AI in employment relationships is so young that reality (practice) might be far ahead of research presented in scientific journals. This issue is even strengthened by the fact that it takes quite some time to get scientific work published. In other words, what do the 23 papers in the analysis reflect in comparison to the rapid changes in TM/HRM and AI in organizations in 2026?

 This raises a more fundamental question: Is this the right time for a literature review? Shouldn’t we be doing more empirical research on the topic in practice?

 The three themes presented, however, are interesting and relevant.

Are the rationale for, and objectives of, the Systematic Review clearly stated?

Partly

Is the statistical analysis and its interpretation appropriate?

Not applicable

If this is a Living Systematic Review, is the ‘living’ method appropriate and is the search schedule clearly defined and justified? (‘Living Systematic Review’ or a variation of this term should be included in the title.)

Not applicable

Are sufficient details of the methods and analysis provided to allow replication by others?

Partly

Are the conclusions drawn adequately supported by the results presented in the review?

Partly

Reviewer Expertise:

Human resource management, talent management, performance management, organizational change, AI, strategy, open science

I confirm that I have read this submission and believe that I have an appropriate level of expertise to state that I do not consider it to be of an acceptable scientific standard, for reasons outlined above.
